# The Long-Term Development of Training, Technical, and Physiological Characteristics of an Olympic Champion in Nordic Combined

**DOI:** 10.3389/fphys.2018.00931

**Published:** 2018-07-13

**Authors:** Vegard Rasdal, Frode Moen, Øyvind Sandbakk

**Affiliations:** ^1^Centre for Elite Sports Research, Department of Neuromedicine and Movement Science, Norwegian University of Science and Technology, Trondheim, Norway; ^2^Department of Education, Norwegian University of Science and Technology, Trondheim, Norway

**Keywords:** endurance training, high-intensity training, mental training, strength training, power, periodization, tapering, concurrent training

## Abstract

Nordic combined requires high technical skills and vertical impulse for the ski-jumping event and aerobic endurance, ski efficiency and finish-sprint abilities to succeed in the subsequent cross-country race. The main aim of this study was to investigate the development of training, technical, and physiological characteristics during the last four seasons preceding the Olympic Games in a Nordic Combined Champion [∼74 kg (63 kg lean-mass)]. During the first season of the 4-year cycle, the development of lower-body muscle-mass and vertical jump velocity was prioritized, after which the emphasis on developing the technical abilities were increased over the following three seasons. While maintaining his vertical velocity in countermovement jump at ∼3 m⋅s^−1^, despite an increase of 7 kg overall body-mass, the participant improved his vertical velocity in sport-specific ski jump imitation with 0.31 m⋅s^−1^ coincidentally with high technical focus, including use of systematic mental training to enhance skill acquisition, and an almost twofold increase of annual imitation jumps in the four-season cycle. Endurance training increased from 462 h⋅season^−1^ in season one to 635 h⋅season^−1^ in season three, which was mainly due to more low-intensity training. Thereafter, endurance training in the Olympic season was reduced by 12% and more focus was placed on quality of each session and sufficient recovery. The highest $\dot{V}$O_2*peak*_ (5.36 L⋅min^−1^ and 72.0 ml⋅kg^−1^⋅min^−1^) was measured in the third season and thereafter maintained, although competition results were further improved toward the Olympics. The amount of moderate- (31.9 ± 2.8 h⋅season^−1^, 43.0 ± 3.9 sessions⋅season^−1^) and high-intensity (28.3 ± 3.1 h⋅season^−1^, 52.3 ± 2.7 sessions⋅season^−1^) endurance training was stable throughout the four-season period, with >65% being performed as skiing or roller ski skating. Development of finish-sprint ability was an important strategy throughout the entire period, and both Olympic gold medals were won in a finish-sprint. Altogether, this study provides unique data from the four-season cycle of a two-time Olympic gold medal winner in Nordic Combined, where high amounts of strength/power and endurance training is successfully combined toward a peak in the Olympic season. This knowledge shows how the combination of long-term endurance and strength/power may be optimized, and generates new hypotheses to be tested in future research.

## Introduction

Nordic Combined (NC) is a challenging Olympic winter sport where the athletes compete in both a ski-jumping event and a cross-country race on the same day (Rasdal et al., 2017). Ski-jumping requires well developed technical abilities, flexibility, high vertical impulse and low body-mass (Schwameder, 2008; Muller, 2009; Sandbakk et al., 2016; Rasdal et al., 2017), in addition to mental awareness and toughness to successfully solve the different phases of a ski jump (i.e., in-run, take-off, transition to flight, flight, preparation for landing, landing). However, the take-off is widely considered most important (Schwameder, 2008; Virmavirta et al., 2009), and a recent study showed vertical velocity achieved in ski jump imitation together with body-mass to account for 70% of the variance in SJ performance in a world cup NC event (Rasdal et al., 2017).

The athletes’ performance in the SJ event results in a proportional time penalty for the following 10-km cross-country skiing pursuit race in the skating style (Rasdal et al., 2017). The ∼25-min cross-country race is performed in varied terrain, in which high aerobic capacity, skiing efficiency and finish-sprint ability is important for performance (Sandbakk et al., 2010; Sandbakk and Holmberg, 2014; Tønnessen et al., 2015; Rasdal et al., 2017).

While there are numerous athletic disciplines where a combination of both endurance and strength/power are required for successful performance, none are as extreme as NC. Hence, the simultaneous development of ski-jumping and cross-country capacity in NC athletes is challenging, and its extreme combination is under-explored. Although only ∼50–60% of the ski-jumping and cross-country specialists’ training is executed by NC athletes in each discipline, they differ only 10–17% in the various laboratory capacities (Sandbakk et al., 2016). Finally, the athlete need to develop mental abilities to optimize both the highly complex technical task in ski-jumping and the physical demanding cross-country event.

The main aim of this study was to investigate the development of training, technical, and physiological characteristics of a NC athlete during a four-season cycle prior to winning two Olympic gold medals at the 2014 Sochi Winter Olympics.

## Materials and Methods

### Participant

The participant (born in 1991) specialized in NC in 2007 and progressively improved performance over the four-season cycle preceding the 2014 Winter Olympics in Sochi where he won two gold medals in NC. See **Table 1** for laboratory capacities in this period that constitutes his four competitive seasons in the World Cup.

**Table 1:** Laboratory capacities determined during the ground preparation phase from 2010–2011 to 2013–2014, as well as immediately after the Olympic Games (Post) in an Olympic Nordic Combined Champion.

		2010–2011	2011–2012	2012–2013	2013–2014	Post
Body-mass	(kg)	66.5	73.2	74.5	73.8	75.3
**Vertical jump velocity**						
Vv_*SQJ*_	(m⋅s^−1^)		2.91	2.94	2.96	3.05
Vv_*CMJ*_	(m⋅s^−1^)		3.04	3.05	3.13	3.10
Vv_*IMIT*_	(m⋅s^−1^)		2.14	2.37	2.34	2.45
**Maximal aerobic power**						
$\dot{V}$O_2*peak*_	(ml⋅kg^−1^⋅min^−1^)	68.8	69.3	72.0	71.0	72.1
	(L⋅min^−1^)	4.58	5.07	5.36	5.24	5.43
**Responses at 4 mmol⋅L^−1^ BLa**						
$\dot{V}$O_2_	(L⋅min^−1^)	3.51	4.01	4.36	4.21	4.31
	(ml⋅kg^−1^⋅min^−1^)	52.8	54.8	58.3	57.0	57.3
	(% peak)	77	79	81	80	79

*^∗^The test with the highest performance level in the period June–October was selected for analysis in the seasons 2010–2011 to 2013–2014. To better reflect the performance level required for the Olympics, also tests immediately post 2013–2014 season was selected (post). Vv_SQJ_, maximum achieved vertical velocity in squat jump; Vv_CMJ_, maximum achieved vertical velocity in countermovement jump; Vv_IMIT_, maximum achieved vertical velocity in imitation jump; $\dot{V}$O_2peak_, peak oxygen uptake from incremental test to exhaustion; BLa, blood lactate concentration; $\dot{V}$O_2_, oxygen uptake.*

The study was approved by the Norwegian Social Science Data Services (NSD), and the participant provided written informed consent to participate in the study.

### Laboratory Testing

General vertical jumps [i.e., countermovement (CMJ) and squat jumps (SQJ)] and sport specific imitation jumps (IMIT) were performed two to three times each season from 2011 to 2012 that was his first season on the national team. Test results from the general preparation phase (GP) was used for descriptive analysis. Equipment and procedures are previously described (Sandbakk et al., 2016; Tønnessen et al., 2016) and, in all jumps, maximum vertical velocity of the center of mass was used for further analysis.

Physiological testing on roller skis was done two to three times each season, and results from GP was used for descriptive analysis. Three or four 5-min submaximal stages to compare physiological response at 4 mmol⋅L^−1^ blood lactate concentration, and an incremental maximal roller ski test to determine $\dot{V}$O_2*peak*_ were performed. Equipment and procedures are previously described (Sandbakk et al., 2010, 2016) and, in all cases, respiratory variables (including determination of $\dot{V}$O_2*peak*_) were calculated according to a previous study (Sandbakk et al., 2016).

One month after the 2014 Winter Olympics, the participant also performed DXA-measurement, from which lean-body-mass is reported.

### Training Monitoring and Systematization of Training Data

The participant recorded his day-to-day training in a digital diary^1^ as previously described (Tønnessen et al., 2016), with all training sessions being systemized and analyzed in Microsoft Office Excel 2016 (Microsoft, Redmond, WA, United States). Endurance sessions were registered using the *modified session-goal approach* (Sylta et al., 2014) as low-intensity (LIT), moderate-intensity (MIT), and high-intensity (HIT) zones and further split into various types of session-categories within each zone, as previously described (Solli et al., 2017). In addition, an own class for recovery, warm-up, and cool-down was defined (WUP).

When speed training was integrated into endurance sessions, 2 min per sprint was registered as speed training. Non-endurance training, such as ski-jumping and strength/power sessions, were registered from the start to the finish of the specific part of the session, including recovery periods between sets. Dry land technique-training was reported as ski-jumping time, and is solely reported as the number of IMITs performed.

Training data are presented annually and divided into different periodization phases, based on key periodization models (Issurin, 2010; Tønnessen et al., 2016) that are slightly modified due to personal communication with the athlete and his coach; Phase 1 (GP1; June–August) and Phase 2 (GP2; September–October) of the General Preparatory Phase, Specific Preparatory Phase (SP; November–December), and Competition Phase (CP; January–March).

Furthermore, in-depth taper analysis of the 2013–2014 Olympic season, including weekly training data over the last 6 weeks and daily training content in the 14 days preceding winning Olympic gold medal, are presented. Of the 6 weeks, the final 2 weeks before winning the first gold medal was defined as peaking phase and the preceding 4 weeks as pre-peaking phase.

### Qualitative Analyses

To track missing data, ensure compliance with the training diary commentaries, and to verify the intensity of different training sessions, two interviews with the participant were conducted during the data-analysis phase of this study. Also two interviews with the participant’s main coach was conducted to gather a qualitative representation of determining factors for the participant’s success. In order to gain an overall understanding of the development of ski-jumping, we performed multi-disciplinary workshops with the authors of this study, his ski-jumping-coach and the mental coach to analyze training logs, tests and competition results, as well as videos and focus during the mental training throughout these seasons.

## Results

**FIGURE 1:**
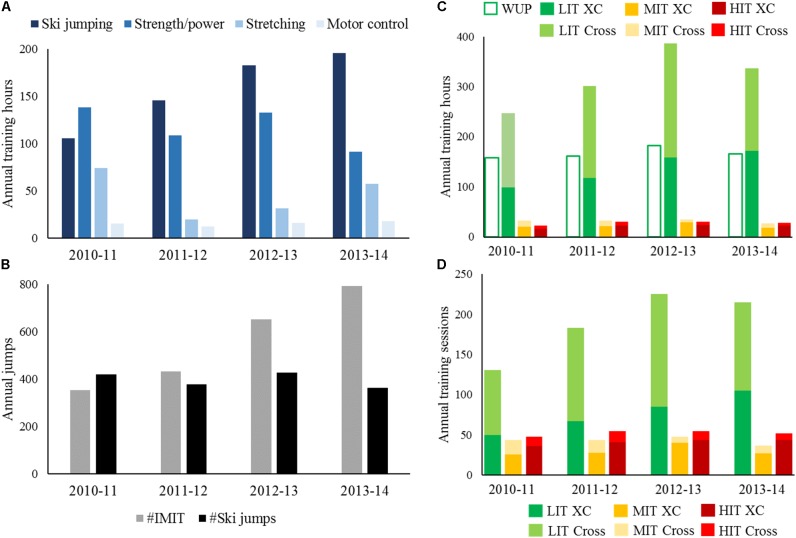
Annual non-endurance training hours **(A)** and number of jumps **(B)**, as well as annual endurance training hours **(C)** and sessions **(D)** from the seasons 2010–2011 to 2013–2014 in an Olympic Champion in Nordic Combined. IMIT, dry-land imitation jump of a ski jump; WUP, time spent for warm-up, cool-down, and recovery; LIT, low-intensity training; MIT, moderate-intensity training; HIT, high-intensity training; XC, cross-country session employing skating technique; cross, endurance session not employing cross-country skating technique.

The participant recorded 804, 824, 1,008, and 950 training hours⋅season^−1^ from 2010–2011 to 2013–2014, distributed across 472, 519, 582, and 585 training sessions. The detailed development of the various training components is depicted in **Figure 1**. The participant gained ∼7 kg of body-mass from 66.5 kg in 2010–2011 to 73.2 kg in 2011–2012, and thereafter stabilized at ∼74 kg (**Table 1**). Lean body-mass was measured shortly after the Olympics to be 63.0 kg.

### Non-endurance

Whereas the amount of general strength/power training varied as a sine wave between 90 and 140 h⋅season^−1^, the amount of ski-jump specific strength/power training and the number of IMITs increased steadily each season (**Figures 1A,B**). Coincidentally, the vertical jump velocity in SQJ and CMJ varied less than 5%, whereas the vertical velocity in IMIT showed a 14.5% increase from 2.14 to 2.45 m⋅s^−1^ in the same period (**Table 2**). The participant reported that the compilation of strength/power sessions in 2010–2011 season was focused toward building lower-body muscle-mass and maximal strength, whereas more high-velocity exercises in ski-jump-specific movement patterns were emphasized from 2011 to 2012. High focus was put on improving ankle-flexibility and hip/core control in the ski-jump-specific movement, and at the same time technically aim to (1) reduce fluctuations of the center of mass in relation to the center of pressure in the in-run and (2) have the center of mass placed vertically above the center of pressure (i.e., lower lever-arm) during the take-off phase.

**Table 2:** Mean session duration and number of monthly training sessions of the different endurance session categories in each intensity zone across seasons and periodization phases from 2010–2011 to 2013–2014 in an Olympic Nordic Combined champion.

	Per season				Mean ± SD of the four-season cycle				2013–2014			
	2010–2011	2011–2012	2012–2013	2013–2014	GP1	GP2	SP	CP	GP1	GP2	SP	CP
**Mean session duration**												
LIT (hrs⋅sess^−1^)	1.9	1.6	1.7	1.6	1.7 ± 0.2	1.7 ± 0.1	1.6 ± 0.1	1.5 ± 0.1	1.6	1.6	1.4	1.3
MIT (min⋅sess^−1^)	44.1	45.2	44.2	44.5	51.2 ± 2.3	48.1 ± 6.1	43.4 ± 4.3	32.2 ± 2.3	53.1	54.6	38.5	29.6
HIT (min⋅sess^−1^)	28.9	34.2	33.5	32.8	35.7 ± 3.5	41.3 ± 3.9	29.8 ± 3.6	27.7 ± 1.0	36.9	39.5	33.4	27.8
**Categories LIT**												
<50 min (sess⋅mth^−1^)	0.2	2.3	2.2	1.5	1.6 ± 1.2	1.5 ± 0.9	2.0 ± 1.3	1.8 ± 0.8	1.0	1.5	2.5	1.7
50–90 min (sess⋅mth^−1^)	1.8	3.6	4.1	6.1	4.2 ± 1.7	2.6 ± 1.2	3.7 ± 1.9	5.3 ± 2.3	6.5	3.4	6.9	7.7
90–120 min (sess⋅mth^−1^)	4.9	3.0	5.3	5.2	6.4 ± 2.4	5.7 ± 1.1	5.0 ± 1.2	2.3 ± 1.4	9.5	6.9	3.4	2.0
>120 min (sess⋅mth^−1^)	3.8	6.2	6.8	4.9	5.0 ± 1.3	6.5 ± 2.9	5.3 ± 0.9	3.7 ± 1.1	3.6	5.4	5.9	3.3
**Categories MIT**												
Continuous (sess⋅mth^−1^)	0.1	0.2	0.1	0.3	0.2 ± 0.2	0.2 ± 0.2	0.1 ± 0.2	0.0 ± 0.0	0.3	0.5	0.5	0.0
<8 min (sess⋅mth^−1^)	1.1	1.1	1.5	0.7	0.7 ± 0.3	1.0 ± 0.6	1.6 ± 0.4	1.7 ± 0.6	0.7	0.5	1.5	1.0
8–15 min (sess⋅mth^−1^)	1.1	0.7	1.5	1.1	1.6 ± 0.3	1.4 ± 0.2	0.9 ± 0.4	0.8 ± 0.4	2.0	1.0	1.0	0.7
>15 min (sess⋅mth^−1^)	0.0	0.3	0.0	0.1	0.2 ± 0.3	0.1 ± 0.2	0.0 ± 0.0	0.0 ± 0.0	0.0	0.5	0.0	0.0
Unspecified (sess⋅mth^−1^)	1.4	1.2	0.9	0.8	2.0 ± 0.8	1.2 ± 0.5	0.5 ± 0.6	0.7 ± 0.6	1.0	1.0	0.0	0.7
**Categories HIT**												
Continuous (sess⋅mth^−1^)	0.3	2.5	2.5	2.4	1.5 ± 0.4	1.4 ± 0.4	3.1 ± 0.7	5.2 ± 0.2	2.0	1.5	2.0	5.0
<3 min (sess⋅mth^−1^)	0.0	0.0	0.1	0.0	0.0 ± 0.0	0.1 ± 0.2	0.0 ± 0.0	0.0 ± 0.0	0.0	0.0	0.0	0.0
3–5 min (sess⋅mth^−1^)	0.5	1.1	1.2	1.2	0.7 ± 0.6	1.4 ± 0.7	1.0 ± 0.6	1.2 ± 0.2	0.7	2.5	1.0	1.0
>5 min (sess⋅mth^−1^)	0.1	0.3	0.2	0.3	0.2 ± 0.4	1.0 ± 0.8	0.1 ± 0.2	0.0 ± 0.0	0.0	1.5	0.5	0.0
Unspecified (sess⋅mth^−1^)	0.4	0.5	0.6	0.3	1.1 ± 0.4	0.2 ± 0.2	0.2 ± 0.2	0.2 ± 0.1	1.0	0.0	0.0	0.3

*GP, general preparatory phase; SP, specific preparatory phase; CP, competition phase; LIT, low-intensity training; MIT, moderate-intensity training; HIT, high-intensity training.*

### Endurance

The participant increased his aerobic capacity by 0.78 L⋅min^−1^ from 2010–2011 to 2012–2013 season, whereas his body-mass-normalized $\dot{V}$O_2*peak*_ was relatively stable across the entire period (**Table 2**). $\dot{V}$O_2_ at 4 mmol⋅L^−1^ blood lactate concentration increased from 77% in 2010–2011 to >80% of $\dot{V}$O_2*peak*_ (**Table 2**).

The participant had a polarized periodization in all seasons, with the overall variation in endurance training volume mainly manipulated by LIT (**Figure 1C**). The amount of MIT and HIT was almost identical in all four seasons, except from a 22% decrease in the amount of MIT from 2012–2013 to 2013–2014 (**Figure 1C**). The main type of intervals for MIT and HIT sessions were in the range of 6–15 min and 3–5 min, respectively (**Table 2**). For all intensities, the average session duration was shorter in SP and CP compared to GP1 and GP2 (**Table 2**). The participant included sprints in nearly all LIT sessions on roller skis and skis, while sprints at the end of some of the MIT/HIT sessions stressed the ability to maintain a well-executed technique when fatigued. Sprint training was included 101, 114, 126, and 129 times⋅season^−1^ in the respective seasons from 2010–2011 to 2013–2014, and was usually performed as approximately ∼5 sprints of 6–8 s.

The amount of endurance training was distributed equally between specific and unspecific training modes (i.e., 45, 44, 47, and 54% cross-country skating in the respective seasons from 2010–2011 to 2013–2014). More than two-thirds of MIT and HIT was performed on skis or roller-skis in all seasons, whereas for LIT 50–60% cross training was performed (**Figures 1C,D**).

Although some training camps throughout the cycle was at altitude > 1,500 m above sea level, no systematic altitude training was performed.

### Tapering

The weekly distribution of training during the final 6 weeks and daily description of the final 2 weeks prior to winning the first gold medal is presented in **Tables 3A,B**. Overall, the training load and distribution between endurance and non-endurance training was similar for all six preceding weeks, except from week −4 and −2 (**Table 3A**), in which total training volume was ∼25% lower in both weeks compared to the other four. The reduction of training load in week −2 was mainly a result of traveling, whereas the reduction in week −4 was a consequence of no ski jumping. The weekly amount of endurance training in week −4 was two-thirds higher compared to the other 5 weeks. From pre-peaking phase to peaking phase, the overall training volume was reduced by 8% whereas endurance training volume was reduced by 25%.

**Table 3:** Weekly training content during the final 6 weeks **(A)** prior to winning two individual gold medals in the 2014 Sochi Winter Olympics, with detailed description of the training performed during the last 14 days **(B)** in an Olympic Nordic Combined champion.

A	Weekly training content during the final 6 weeks prior to gold medal					

Week	Non-endurance hours (sessions)	*SJ hours (sessions)*	Endurance hours (sessions)	*XC MIT hours (sessions)*	*XC HIT hours (sessions)*	Total hours (sessions)
−6	10.3 (6)	*7.3 (5)*	8.7 (6)	*0.0 (0)*	*0.8 (2)*	18.9 (12) Two competitions
−5	10.6 (6)	*8.2 (5)*	10.2 (7)	*0.0 (0)*	*1.5 (3)*	20.8 (13) Three competitions
−4	1.8 (3)	*0.0 (0)*	14.8 (8)	*0.8 (1)*	*0.0 (0)*	16.7 (11)
−3	11.4 (9)	*6.3 (5)*	8.9 (5)	*0.0 (0)*	*1.2 (2)*	20.4 (14)
−2	7.1 (4)	*3.9 (3)*	7.9 (5)	*0.0 (0)*	*0.8 (2)*	15.0 (9)
−1	11.5 (5)	*6.7 (4)*	9.1 (7)	*0.7 (2)*	*0.0 (0)*	20.6 (12)
0	*Individual gold medal, Olympic Winter Games 2014*					

**B**	**Daily training content during the last 2 weeks prior to gold medal**					

**Day**	**AM**			**PM**		

−14	*Rest day*					
−13	1.5 h strength/power^∗^			1.5 h LIT with 4 × 6–8 s sprints, XC		
−12	0.75 h LIT, running + 0.25 h flexibility			*Travel*		
−11	*Travel day*					
−10	1.5 h LIT, running			1 h dry land technique session		
−9	2 h SJ^*c*∗^			5 × 3 min HIT^d^, XC		
−8	2 h SJ^*c*∗^			1 h LIT, XC		
−7	2 h SJ^*c*∗^			1 h LIT, XC		
−6	1.5 h strength/power^∗^			0.3 h LIT, running + 0.3 h flexibility		
−5	0.5 h LIT, running			1.25 h LIT, running		
−4	0.3 h LIT, running + 0.7 h flexibility			0.25 h flexibility		
−3	2.5 h SJ^*c*∗^			5 × 7 min MIT^d^, XC		
−2	2.5 h SJ^*c*∗^			1.25 h LIT, XC		
−1	2 h SJ^*c*∗^			1 h LIT with 3 × 8 s sprints, XC		
0	*Individual gold medal, Olympic Winter Games 2014*					

*^c^Official ski jumping training in relation to competition. ^d^MIT and HIT sessions normally included 20–40 min of LIT as warm-up and 15–30 min LIT as cool-down. SJ, ski jumping session; XC, cross-country skiing employing skating technique; technique session, exercises designed to imitate ski jumping on dry land; LIT, low-intensity training; MIT, moderate-intensity training; HIT, high-intensity training. ^∗^Time of non-endurance sessions is from start to the end of session, including warm-up and recovery between sets.*

### Qualitative Assessment

The participant was involved in multiple of sport disciplines until the start of high-school, when he decided to specialize in NC at the age of 16. Since then, he had a close and well-functioning working-alliance with the same, high-level coach, with all training directed toward sport-specific goals. He was also part of a well-functioning training group that included two of the world’s best NC athletes throughout the entire period. Here, regular team processes and daily training provided the possibility to develop and train at the highest level. During this period, he used mental training systematically to improve ski-jump-technique by, e.g., developing an automatized awareness of in-run position and balance and to optimize the take-off dynamics, especially in stressful situations in the hill. Although the participant quickly improved technical skills in dry-land training, the ability to translate this to the ski-jumping hill was more gradual. Hence, his ability to perform on top in important competitions were gradually improved and optimized toward the Olympics.

Sequencing of sessions throughout the seasons was based on the competition format of the sport, i.e., with strength/power and ski-jump sessions performed early in the day and endurance sessions in the afternoon with 2–4 h in between (when performed on the same day). For seasonal periodization, the GP1 contained relatively more focus on strength/power and high load of endurance training, whereas the focus on key development sessions (i.e., SJ sessions, and intervals on roller skis/skis) increased from GP2 toward CP.

## Discussion

The present study investigated the development of training, technical, and physiological characteristics during the last four seasons preceding the Olympic Winter Games in an Olympic NC Champion. After an initial focus of increasing lower-body muscle-mass and vertical jump velocity, the participant had a greater emphasis on technical sessions over the following three seasons. At the same time, the athlete was included in a group of world-class NC athletes and systematic mental training to enhance skill acquisition was included. After a progressive increase of endurance training over the first three seasons, this was reduced by 12% in the Olympic season. While maintaining his CMJ vertical jump velocity at ∼3 m⋅s^−1^, despite an increase of 7 kg overall body-mass, the participant improved his vertical jump velocity of sport-specific IMITs with 0.31 m⋅s^−1^ and $\dot{V}$O_2*peak*_ with ∼0.8 L⋅min^−1^ coincidentally with an almost twofold increase of annual IMITs and an increase of ∼200 annual endurance hours in the four-season cycle. An emphasis on improving finish-sprint ability in cross-country skiing was present in all seasons, a determining factor since both Olympic gold medals were won in the finish-sprint. Tapering toward the Olympic included a 25% reduction in endurance training volume and an 8% increase in non-endurance training from pre-peaking to peaking phase.

### Non-endurance

Already in 2011, the athlete had reached a world-class vertical jump velocity of ∼2.9 m⋅s^−1^ in CMJ and SQJ (Tønnessen et al., 2016; Rasdal et al., 2017). This was likely a consequence of the strength/power focus in the 2010–2011-season, compiled toward muscle hypertrophy and maximal lower-body strength. Thereafter, strength/power sessions were focused more toward high-velocity exercises with ski-jump-specific movement pattern. This shift of exercise content, with more specificity when the level of strength was sufficient, also allowed for maintaining his jump capacity along with the large increase of endurance training that often induce negative influence on power development (Nader, 2006; Wilson et al., 2012; Baar, 2014). Coinciding the increase in the number of IMITs, ski-jump-specific vertical jump velocity improved with ∼15%, indicating that the execution of this technical skill was not negatively influenced by concurrent endurance training. Overall, it may be beneficial for NC athletes to improve strength and vertical impulse at an early phase, followed by a greater focus on high-velocity exercises in a ski-jump-specific movement pattern when increasing the annual endurance load.

With an in-run speed of 85–95 km⋅h^−1^ and less than 0.35 s to complete the take-off (Muller, 2009), the athlete relies on an automated and technically optimized take-off pattern. Improved ankle-flexibility and hip/core control enabled our participant to solve the task (see description in the “Results” section) well during dry-land training and testing already early in the Olympic cycle, but it required longer time to transfer this skill to ski-jumping in the hill. Here, systematic mental training to enhance skill acquisition supported the technique training, which was likely a crucial factor for the participant’s success at the Olympic Games. Since we do not have good quantitative measurement of these aspects of the ski-jump technique, future studies should strive to develop and validate such measurements both for laboratory and field testing.

### Endurance

The polarized intensity model and overall endurance volume of ∼560 h⋅season^−1^ in the Olympic season is similar to earlier reported values from successful seasons in NC athletes (Tønnessen et al., 2016). The amount of endurance training was, however, increased by an average of 87 h⋅season^−1^ over the initial three seasons, having a peak of 635 h⋅season^−1^, followed by a reduction in the Olympic season. While this is clearly lower than top-level cross-country skiers who progress their training up to ∼8–900 annual endurance training hours (Sandbakk and Holmberg, 2014; Solli et al., 2017), NC athletes train more hours than cross-country skier when including their ski-jumping training (Sandbakk et al., 2016). However, the annual training cannot be directly compared between the two winter sports as the different loads of cross-country versus ski-jumping training and the subsequent risk of overreaching must be considered differently. Nevertheless, the current study indicates that a progressive increase in endurance training load during an Olympic cycle followed by a reduction in the peak season may be beneficial for overall long-term development in NC.

The annual increase in endurance training load until the 2012–2013 season, coupled with 7 kg increase in body-mass, led to a gradual increase of $\dot{V}$O_2*peak*_ up to 5.36 L⋅min^−1^ and 72.0 ml⋅kg^−1^⋅min^−1^, which is within previously reported benchmark values in world-class NC athletes (Tønnessen et al., 2015; Sandbakk et al., 2016; Rasdal et al., 2017). In the 2012–2013 season, ∼80% of MIT and HIT sessions (compared to 70% before) was performed in skating, which most likely contributed to the increased utilization of $\dot{V}$O_2*peak*_ at 4 mmol⋅L^−1^ of blood lactate, as well as a further increase in roller ski $\dot{V}$O_2*peak*_ that season. The subsequent reduction of endurance training load from 2012–2013 to 2013–2014 was partly compensated by more skating also in the LIT zone, and might have led to maintenance of endurance capacity in the Olympic season. However, the lower endurance load may have reduced overall fatigue and thus improved the quality in the non-endurance sessions as well, enabling a further development of the vertical jump velocity in the Olympic season (measured after the 2013–2014 season). This may overall suggest that the reduction was beneficial for development of ski-jumping performance, and thus, the overall NC performance.

### Tapering

The tapering strategy of 25% reduction in endurance training load from the pre-peaking phase to the peaking phase is somewhat higher than previously found in successful cross-country skiers and biathletes (Tønnessen et al., 2014; Solli et al., 2017), and is more than the 20% reduction recommended for achieving a tapering effect (Bosquet et al., 2007). Overall training load, however, was decreased by only 8% as non-endurance load was increased from pre-peaking to the peaking phase. Tønnessen et al. (2014) speculated in their study that the lower reduction in training volume found among elite cross-country skiers compared to what is suggested by the literature could be ideal in sports with a dense competition schedule. However, NC athletes are also dependent on ski-jumping facilities to be able to jump, and thus logistics govern much of their training plan. This may also explain the three-phase tapering format in the participants’ endurance training load, where a 45% increase from week −5 to −4 was coupled with no ski-jumping in week −4. How to taper for an optimal ski-jumping performance has not previously been researched, and its delicate interplay with endurance training to achieve optimal NC performance sorely needs to be further investigated in future group studies.

## Conclusion

Our study provides unique data from the four-season cycle of a two-time Olympic gold medal winner in NC. The participant focused on increasing lower-body muscle strength and vertical jump impulse early in the cycle, followed by increased emphasis on technical ski-jumping sessions and inclusion in a high-level training group. Here, improved ankle-flexibility and hip/core control, together with systematic mental training, gradually enabled our participant to solve the technical task in the hill. A progressive increase of low-intensity endurance training over the three first seasons was followed by reduced endurance training volume, but with a higher degree of specific training in the Olympic season to enable greater quality in each session and to trigger surplus in developing ski-jumping performance. Improving finish-sprint ability was emphasized in all seasons, and was a determining factor for winning both Olympic gold medals. Peaking toward the Olympics included an overload of endurance training in the pre-peaking phase before a 25% reduction in the peaking phase. Consequently, non-endurance training was increased from pre-peaking to the peaking phase and the overall training was not reduced more than 8%. Altogether, this study provides insight into how the combination of long-term endurance and strength/power training may be optimized, and generates new hypotheses to be tested in future group studies. In particular, detailed description and analysis of non-endurance training is lacking in the majority of studies on concurrent and strength/power sports. We thus encourage future studies to investigate the training load of strength/power, both as an isolated stimuli, and concurrently to endurance training.

## Author Contributions

VR, FM, and ØS designed the study, contributed to interpretation of the results, and contributed to the final manuscript. VR performed the data collection. VR and ØS performed the data and statistical-analysis and wrote the draft manuscript.

## Conflict of Interest Statement

The authors declare that the research was conducted in the absence of any commercial or financial relationships that could be construed as a potential conflict of interest.

## Footnotes

1 www.olt-dagbok.net
